# Impact of imatinib on the pharmacokinetics and *in vivo *efficacy of etoposide and/or ifosfamide

**DOI:** 10.1186/1471-2210-7-13

**Published:** 2007-10-27

**Authors:** Keyvan Rezaï, François Lokiec, Isabelle Grandjean, Sophie Weill, Patricia de Cremoux, Vincent Bordier, Richard Ekue, Mickael Garcia, Marie-France Poupon, Didier Decaudin

**Affiliations:** 1 Department of Pharmacology Oncology, Centre René Huguenin, Saint-Cloud, France; 2 Unit of Animal experiments, Research Section, Institut Curie, Paris, France; 3 Department of tumor Biology, Institut Curie, Paris, France; 4 Department of Clinical Hematology, Institut Curie, Paris, France; 5 FRE 2584, Section de Recherche, Institut Curie, Paris, France; 6 UMR144 CNRS/Institut Curie, Paris, France

## Abstract

**Background:**

Using a human small cell lung cancer (SCLC) xenografted in *nude *mice, we have previously reported enhanced tumor growth inhibition following chemotherapy in combination with imatinib (STI571). We therefore investigated the *in vivo *impact of imatinib on the pharmacokinetics and efficacy of chemotherapy.

**Methods:**

Two different human tumors were used: SCLC6 small cell lung cancer xenografted in *nude *mice, and LY-3 EBV-associated human B-cell lymphoma xenografted in SCID mice. Plasma, urine, and fecal concentrations of etoposide (VP16) were determined by a validated high performance liquid chromatography method. Plasma concentrations of ifosfamidewere determined by a validated gas chromatography assay with nitrogen-phosphorus detection.

**Results:**

Slight tumor growth inhibition was induced by imatinib administered alone in one *in vivo *EBV-associated B-cell lymphomatous xenograft. In contrast, an increase of the chemotherapy-induced antitumor effect was observed in the lymphoma model but not in a small cell lung cancer model when mice bearing human xenografted tumors were treated concomitantly by imatinib and chemotherapy. This antitumor effect was not influenced by concomitant administration of fluconazole. The AUC0-3 h (Area Under the concentration-time Curve) of etoposide was increased when mice were treated with etoposide + imatinib due to decreased fecal excretion. In contrast, imatinib did not appear to influence the urinary excretion of etoposide, and concomitant administration of the CYP3A4 inhibitor, fluconazole, with imatinib did not modify the pharmacokinetics of etoposide plus imatinib alone.

**Conclusion:**

Altogether, these results therefore justify further prospective phase I and II clinical trials with combinations of etoposide-based chemotherapy and imatinib in patients with certain cancers, such as malignant lymphoma, with careful toxicologic monitoring.

## Background

The tyrosine kinase inhibitor imatinib (STI571), belonging to the 2-phenylaminopyrimidine class, selectively inhibits BCR/ABL [1], PDGFR ("Platelet-Derived Growth Factor receptor"), c-kit [2], and c-fms ("Macrophage colony-stimulating factor receptor")[3] kinase activity. As imatinib is commonly used without chemotherapeutic agents, few reports have evaluated the therapeutic effect of concomitant administration of imatinib and chemotherapy either in mice xenografted tumors or cancer patients. Using a human small cell lung cancer (SCLC) xenograft in *nude *mice, we previously reported enhanced tumor growth inhibition following chemotherapy (etoposide + ifosfamide or topotecan) in combination with imatinib and showed that this effect was not dependent on c-kit expression level [4]. The increase of conventional antineoplastic agent-induced tumor growth inhibition was also exclusively observed when imatinib and chemotherapy were administered concomitantly. Various mechanisms can be proposed to explain this effect, namely (1) an increase of drug uptake by imatinib-induced reduction of tumor interstitial fluid pressure and an increase of transcapillary transport [5], (2) inhibition of tumor angiogenesis combined with the antitumor effect of chemotherapy [6-8], and (3) certain intracellular events induced by the two concomitant therapeutic modalities that remain to be determined.

However, our *in vivo *experiments showed a higher toxicity of combined imatinib and chemotherapy than for imatinib or chemotherapy alone, exclusively when both treatments were administered concomitantly. Various mechanisms for the toxicity of combined therapy can be proposed, particularly pharmacokinetic interactions between imatinib and chemotherapeutic agents that could also explain the increase of chemotherapy-induced tumor growth inhibition, as numerous data have shown that imatinib induces cytochrome p450 inhibition and that this inhibition decreases elimination of chemotherapeutic agents such as etoposide, ifosfamide, and topotecan [9-12]. In order to explore these pharmacokinetic interactions, we therefore investigated the *in vivo *impact of imatinib on the pharmacokinetics of chemotherapy and its efficacy on two different human cancers xenografted into immunodeficient mice: a SCLC [4] and an EBV-associated B-cell lymphoproliferation [13]. We showed that imatinib significantly increases the AUC (Area Under the concentration-time Curve) of etoposide in mice via a decrease of its fecal excretion. Finally, in one of the two xenografted models used, we confirmed the imatinib-induced increase of tumor growth inhibition after chemotherapy with agents such as etoposide (VP16) and gemcitabine. These results therefore support the initiation of further prospective phase I and II clinical trials combining etoposide-based chemotherapy and imatinib in cancer patients, such as malignant lymphoma patients, with careful toxicologic monitoring.

## Methods

### *In vivo *experiments in immunodeficient mice bearing human tumors

Female *nude *or SCID mice, weighing 20 g to 30 g, 6–8 weeks old, were bred in the animal facilities (Institut Curie, Paris, France), maintained under specific pathogen-free conditions with artificial lighting (12-hour light/12-hour dark cycle) and fed with a regular diet and water *ad libitum*. The care, housing, and handling of the mice were performed in accordance with the recommendations of the French Ethics Committee and under the supervision of authorized investigators. For curative therapeutic trials, the tumor-bearing mice were randomly divided into equivalent groups of 6 to 8 animals and mice were treated at different times after transplantation.

Two different human tumors were used: the SCLC6 small cell lung cancer xenografted in *nude *mice [4], and the LY-3 EBV-associated human B-cell lymphoma xenografted in SCID mice [13]. STI571 (gift from Novartis Pharma SAS, Rueil-Malmaison, France) was diluted in 150 μl of H2O and administered at a total dosage of 70 mg/kg per day in one intraperitoneal injection on different days, as indicated. Etoposide (VP16)(Pierre Fabre, Boulogne, France) and ifosfamide (Baxter, Paris, France) were diluted in 200 μl of 0.9% sodium chloride and administered at a dose of 12 mg/kg and 90 mg/kg, respectively, in one daily intraperitoneal injection on days 1 to 3 of treatment. Fluconazole (PFIZER Paris, France) was diluted in 400 μl of water and administered at a total dosage of 40 mg/kg per day in one intraperitoneal injection on days 1 to 3 of treatment. Gemcitabine (Lilly France SAS, Suresnes, France) was diluted in 150 μl of 0.9% sodium chloride and administered in one weekly intraperitoneal injection at a dosage of 60 mg/kg per day. The control group received 0.9% sodium chloride injections according to the same schedule as experimentally treated mice.

All mice were weighed once weekly. Tumor growth was monitored by measuring two perpendicular diameters with calipers. Tumor volume (V) and relative tumor volume (RTV) were calculated as follows:

V = *a*^2 ^× *b*/2,

where *a *is the width (large diameter) and *b *the length (small diameter) of the tumor in millimeters.

RTV = V_x_/V_i_,

where V_x _is the mean tumor volume in cubic millimeters at any given time and V_i _is the mean initial tumor volume in cubic millimeters at the start of treatment [14]. Mice were ethically sacrificed when the tumor volume reached 2,500 mm^3 ^in the control group.

### Pharmacokinetics of etoposide (VP16) and/or ifosfamide in mice

In order to evaluate pharmacokinetic interactions between STI571 (imatinib) and chemotherapeutic agents (etoposide and ifosfamide), various concentrations of the two cytotoxic agents were measured. Plasma, urine, and fecal concentrations of etoposide were determined by a validated high performance liquid chromatography (HPLC) method with U.V. detection [15]. Standard samples were prepared from a stock solution of 1000 μg/ml etoposide which was added to drug-free pooled plasma or urine. The analytes were extracted from plasma, urine and feces by liquid-liquid extraction into aqueous phase with dichloromethane. All 24-hour fecal samples were weighed and homogenized with 1 ml of water. Plasma concentrations of ifosfamidewere determined by a validated gas chromatography assay (GC) with nitrogen-phosphorus detection [16]. The analytes were extracted from plasma and urine by liquid-liquid extraction into aqueous phase with ethyl acetate.

### Evaluation of c-kit mRNA expression

Total RNA extraction and cDNA synthesis were performed as previously described [17]. Briefly, total RNA was extracted from crushed tumor samples (SCLC 6, SCLC 61, SCLC 74 and SCLC 108) by RNA plus^® ^kit (Bioprobe, France). One μg of total RNA was reverse transcribed in a final volume of 20 μL containing 1× reverse transcriptase buffer [500 mM of each deoxynucleotide triphosphate, 3 mM MgCl2, 75 mM KCl, and 50 mM Tris-HCl (pH 8.3)], 10 units of RNase inhibitor (Promega, Madison, WI), 10 mM DTT, 50 units of Superscript II RNAse H-reverse-transcriptase (Life Technology, Inc), and 1.5 mM random hexamers (Pharmacia, Uppsala, Sweden). The reaction mix was then incubated at 42°C for 30 min.

C-kit transcripts were quantified using real-time quantitative reverse transcription-PCR assays. Primers and probes were chosen with the assistance of Primer Express software (Applied Biosystems, Foster City, CA-IC). The nucleotide sequence was then blasted against dbEST and nr (the non-redundant set of the GenBank, EMBL and DDBJ database sequences) to confirm the total gene specificity of the nucleotide sequences chosen as primers at probes. Each primer couple was positioned in different exons (i.e. exons 20–21 for c-kit gene) of the gene in order to avoid amplification of contaminating genomic DNA. PCR reactions were performed using an ABI Prism 7700 Sequence Detection System (Applied Biosystems, Foster City, CA-IC) and Core Reagent Kit (Eurogenetec, Belgium). Real-time detection was performed using oligonucleotide probes containing a fluorescent dye at its 5'-end and a quencher at its 3'-end (for c-kit mRNA quantification).

Fluorescent probes were synthesized by Applied Biosystems, and primers were synthesized by Invitrogen (Paisley, UK). The nucleotide and probe sequences are as follows: c-kit: upper primer 5'-aagcagatttcagagagcacca-3', lower primer 5'gctgccgacagaattgatcc-3' and probe 5'actccaacttagcaaactgcagccccaa-3'. Transcripts of TBP (TATA box-binding protein)[18] were also quantified as endogenous RNA of reference genes to normalize c-kit expression.

The thermal cycling conditions comprised an initial denaturation step at 95°C for 10 min, then 40 cycles at 95°C for 15 sec and an annealing temperature depending upon the target. For c-kit quantification, a calibration curve for mRNA expression was generated using serial dilutions of L174 human colon carcinoma cell line mRNA expressing high levels of c-kit mRNA to assess PCR efficiency. All measurements were performed under blind conditions and in duplicate. Two negative "non-template" controls were included in each amplification run.

Results were expressed as N-fold differences in target gene expression relative to the reference gene (either TBP) and the calibrator was called "Ntarget". It was determined as follows: N target = E^(ΔCt sample-ΔCt calibrator)^, where the ΔCt values of the sample and calibrator were determined by subtracting the average Ct values of the target gene from the average value of the reference gene (TBP) and E is the efficiency of PCR measured using the slope of the calibration curve. For c-kit mRNA quantification, the positive control, corresponding to a case of uveal melanoma exhibiting strong c-kit overexpression, was assessed with SCLC samples.

### Statistical analysis

Statistical analysis of variance (ANOVA test) was performed to assess the difference between AUC_0–3 h _(Area Under the concentration-time Curve) of etoposide in the various treatments. A Mann-Whitney U-test was used to assess the *in vivo *effect of the various treatments on the growth of xenografted tumors in *nude *or SCID mice. U values were considered significant when the probability of a difference was less than or equal to 0.05. Values were considered significant when the probability of a difference was less than or equal to 0.05.

## Results

### Impact of STI571 on the *in vivo *xenografted tumor growth of etoposide (VP16) chemotherapy

**Figure 1:**
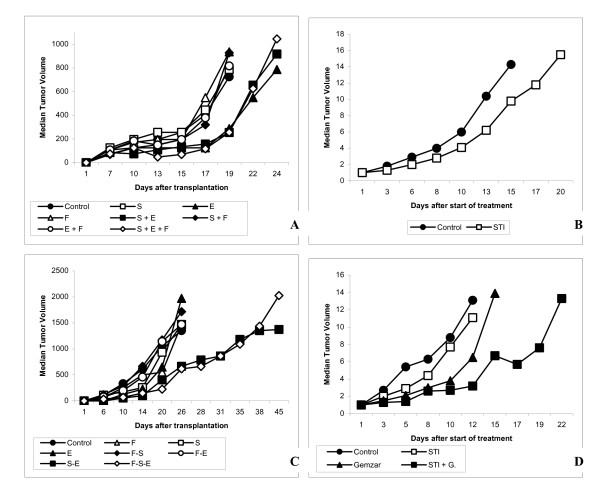
**Antitumor activity of imatinib with or without chemotherapy in two human xenografted tumors**. (A)(C). Both xenografted SCLC6 (A) and LY-3 (C) tumors were treated by etoposide (VP16) alone (E) at a dosage of 12 mg/kg in one daily intraperitoneal injection on days 1 to 3 (▲), etoposide (VP16) and STI571 (E + S) administered by one daily intraperitoneal injection at a dose of 70 mg/kg on days 1 to 3 (■), etoposide (VP16) and fluconazole (E + F) at a dosage of 40 mg/kg in one daily intraperitoneal injection on days 1 to 3 (○), or etoposide (VP16) with STI571 and fluconazole (E + S + F)(◇). All other groups included STI571 alone (S)(□), fluconazole alone (F)(△), and STI571 + fluconazole (S + F)(◆) and 0.9% NaCl (●). (B) Mice bearing LY-3 tumors were treated by one (□) daily intraperitoneal injection of STI571 (S) at a dose of 70 mg/kg from day 1 until sacrifice of the animals. (D) Xenografted LY-3 tumors were treated by gemcitabine at a dosage of 60 mg/kg by one weekly intraperitoneal injection, with (G + S)(■) or without (G)(▲) STI571 administered by one daily intraperitoneal injection at a dose of 70 mg/kg from day 1 until sacrifice of the animals. Mice treated by STI571 alone (S) are indicated by (□). All control groups received injections of 0.9% NaCl (Control)(●). Tumor growth was evaluated by measuring the relative tumor volume (RTV), as described in "Materials and Methods". A Mann-Whitney *U *test was used to assess the effects of treatments on xenografted tumor growth.

SCLC6-bearing *nude *mice received intraperitoneal injections of etoposide (12 mg/kg) on days 1–3, with or without STI571 administered on days 1–3 by intraperitoneal injections at a total dose of 70 mg/kg daily, and with or without intraperitoneal injections of fluconazole (40 mg/kg/d) on days 1–3. Each treatment was initiated on day + 6 after tumor transplantation. We observed a brief but non-significant inhibition of SLCL6 tumor growth after etoposide administration compared to the control group (p = NS), and this inhibition was not increased by concomitant injections of etoposide + STI571 or etoposide + STI571 + fluconazole (Figure 1A).

As previously performed for the SCLC6 tumor (Decaudin IJC), we first evaluated the antitumor effect of STI571 alone in the SCID mice xenografted LY-3 lymphomatous tumors. Mice bearing tumors measuring approximately 60 mm^3 ^were treated from day 1 until sacrifice of the animals by an intraperitoneal injection of STI571 at a dose of 70 mg/kg. A slight but non-significant tumor growth inhibition was temporarily observed for a few days after initiation of treatment (Figure 1B). Thereafter, SCID mice bearing LY-3 tumors were treated by intraperitoneal injections of etoposide (12 mg/kg) on days 1–3, with or without intraperitoneal injection of STI571 at a dose of 70 mg/kg on days 1–3, and with or without intraperitoneal injections of fluconazole (40 mg/kg/d) on days 1–3. Each treatment was initiated at day + 6 after tumor transplantation. We observed a spectacular enhancement of etoposide-induced tumor growth inhibition by concomitant administration of STI571 (p < 0.05), and this inhibition was not enhanced by concomitant administration of fluconazole (Figure 1C). Moreover, we observed three early deaths in the combined etoposide + STI571 group, but not in the etoposide + STI571 + fluconazole group.

Finally, in order to confirm the value of combining STI571 with other chemotherapy modalities, mice bearing LY-3 tumors measuring approximately 60 mm^3 ^received weekly intraperitoneal injections of gemcitabine (60 mg/kg), with or without intraperitoneal injections of STI571 at a total dose of 70 mg/kg daily from day 1 until sacrifice of the animals. We observed an enhancement of gemcitabine-induced tumor growth inhibition by concomitant and continuous administration of STI571 (p < 0.01)(Figure 1D).

### Impact of STI571 on the pharmacokinetics of combined etoposide (VP16) and ifosfamide chemotherapy regimen in mice

**Figure 2:**
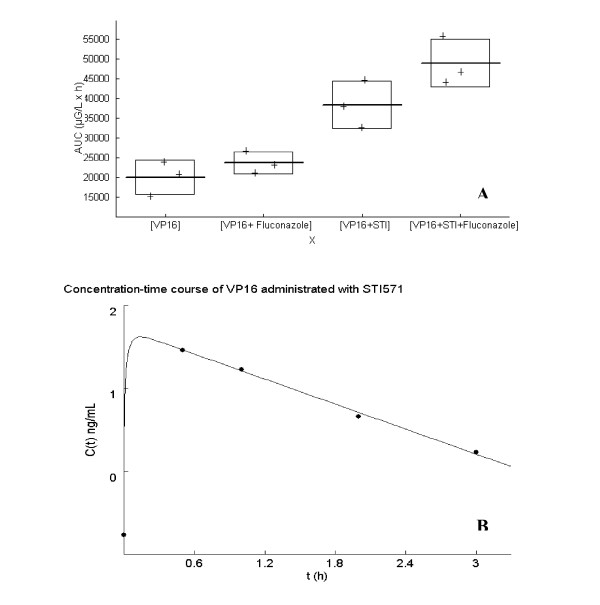
**(A) Mean AUC_0–3 h _of etoposide (VP16) in CD1 mice**. Area Under Curve concentration versus time of etoposide (VP16) was calculated between base time (0) and 3 hours after administration of etoposide alone or etoposide plus fluconazole or etoposide plus imatinib or etoposide with imatinib and fluconazole. **(B) Concentration-time curve of etoposide (VP16) administered with STI571**. After administration of etoposide (VP16) with STI571, concentrations of VP16 peaked at 30.9 ± 2.1 ng/mL, followed by a bi-exponential decline. **(C) Graphical analysis between calculated concentrations and observed concentrations of etoposide (VP16)**.

To study the impact of STI571 on the *in vivo *pharmacokinetics of chemotherapy, C57 Black6 mice were treated on days 1 to 3 by concomitant intraperitoneal injections of etoposide (12 mg/kg/d) and ifosfamide (90 mg/kg/d), with or without STI571 (70 mg/kg/d), and serum was collected from an eye vein on days 1 and 3, 0, 1, 3 and 6 hours after injections. We observed an increase of the Cmax of both etoposide and ifosfamide on days 1 and 3 when mice were treated by chemotherapy combined with STI571 compared to chemotherapy alone (Table 1). To confirm this result and to focus on etoposide pharmacokinetics, CD1 mice were treated on days 1–3 by intraperitoneal injections of 12 mg/kg/d etoposide, with or without intraperitoneal 70 mg/kg/d of STI571. Samples were collected on days 1 and 3, 0.5, 1, 2 and 3 hours after injections. We observed a significant increase of the mean AUC_0–3 h _(Area Under the concentration-time Curve) of etoposide (17900 μg/l × h) when mice were treated with etoposide + STI571 (60800 μg/l × h)(p = 0.025)(Figure 2A).

**Table 1:** Mean Cmax of etoposide (VP16) and ifosfamide administered alone or with imatinib

	***Ifosfamide***	***Ifosfamide *+ imatinib**
***Mean Cmax***	9 +/- 4.5 μg/ml	14.4 +/- 6.4 μg/ml
	***Etoposide***	***Etoposide *+ imatinib**
***Mean Cmax***	8.6 +/- 4.1 μg/ml	42.7 +/- 37.6 μg/ml

In order to evaluate the role of CYP3A4 on the pharmacokinetic interaction between etoposide and STI571, CD1 mice were treated on days 1–3 by intraperitoneal injections of etoposide (12 mg/kg/d), with or without STI571 (70 mg/kg/d), and with or without the CYP3A4 inhibitor, fluconazole (40 mg/kg/d). The samples were collected on days 1 and 3, 0.5, 1, 2 and 3 hours after injections. A summary of etoposide pharmacokinetic parameters (mean) is presented in Table 2. We observed a highly significant increase of the mean AUC_0–3 h _of etoposide (19970 μg/l × h) when mice were treated with etoposide + STI571 + fluconazole (48960 μg/l × h), as compared to either etoposide + STI571 (38380 μg/l × h) or etoposide + fluconazole (23680 μg/l × h) combinations (p = 0.0003)(Figure 2A). In both experiments, after administration of VP16 with STI571, VP16 concentrations peaked at 30.9 ± 2.1 ng/mL, followed by a bi-exponential decline (Figure 2B). VP16 concentrations reached 46.5 ± 6.4 ng/mL when VP16 was administered with STI571 and fluconazole. In both cases, the pharmacokinetic curve was best described as bi-exponential as demonstrated by the concentration-time curve of VP16 administered with STI571 (Figure 2C).

**Table 2:** Pharmacokinetic parameters of Etoposide (VP16) administered alone or in combination

	**Etoposide**	**Etoposide + fluconazole**	**Etoposide + imatinib**	**Etoposide + fluconazole + imatinib**
**T 1/2 (hours)**	0.24	0.23	0.35	0.49
**AUC (μg/L × h)**	19970	23680	38380	48960
**Clearance (ml/h)**	0.62	0.51	0.32	0.25

**Figure 3:**
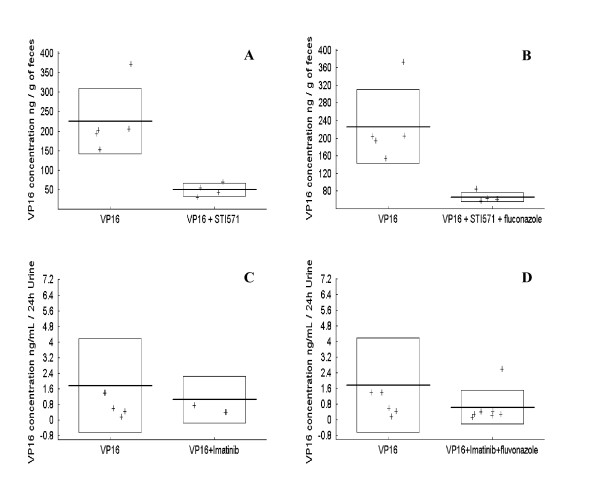
**Fecal and urine excretion of etoposide in CD1 mice**. Etoposide (VP16) levels were determined in feces (ng of etoposide/g of feces) when mice were treated with etoposide alone (A, B) or in combination with imatinib (B) or in combination with imatinib and fluconazole (B). Etoposide (VP16) levels were determined in urine (ng of etoposide/24 hours of urine) when mice were treated with etoposide alone (C, D) or in combination with imatinib (C) or in combination with imatinib and fluconazole (D).

To explain the impact of STI571 on the pharmacokinetics of etoposide, we studied the liver and kidney clearance of etoposide by assaying the chemotherapeutic agent in urine and feces of CD1 mice treated on day 1 by one intraperitoneal injection of etoposide (12 mg/kg/d) with or without STI571 (70 mg/kg/d). Samples were collected for 24 hours after injection. We observed a significant decrease of fecal excretion of etoposide when mice were treated with either etoposide + STI571 (p = 0.005)(Figure 3A) or etoposide + STI571 + Fluconazole (p = 0.003) (Figure 3B). In contrast, no significant impact of STI571 was observed on the renal clearance of etoposide, with (Figure 3C) or without fluconazole (Figure 3D).

### Evaluation of c-kit mRNA expressions

The level of c-kit mRNA expression was quantified and compared to the level of mRNA expression of a known c-kit protein overexpressing melanoma. As previously reported [4], the two SCLC6 and LY-3 xenografts express very low levels of c-kit mRNA (data not shown).

## Discussion

In conclusion, we have shown that imatinib increased the AUC of etoposide in mice and that this effect was mediated by a reduction of its fecal excretion. In contrast, imatinib did not appear to influence the urinary excretion of etoposide, probably due to sample collection conditions, and concomitant administration of the CYP3A4 inhibitor fluconazole with imatinib did not modify the pharmacokinetics of etoposide plus imatinib alone. We also demonstrated a slight tumor growth inhibition induced by imatinib administered alone in one *in vivo *EBV-associated B-cell lymphomatous xenograft, and this effect was observed despite the absence of c-kit receptor mRNA expression. Finally, when mice bearing human xenografted tumors were treated concomitantly by imatinib and chemotherapy, an increase of the chemotherapy-induced antitumor effect was observed in the lymphoma model but not in a small cell lung cancer model, and this antitumor effect was not influenced by concomitant administration of fluconazole.

Our *in vivo *B-cell lymphoma studied by RT-PCR was negative for c-kit mRNA expression. This result was concordant with that reported in the literature, where c-kit expression, studied by RT-PCR or immunohistochemical methods, was mainly observed in multiple myeloma, CD30-positive anaplastic large cell lymphoma, and Hodgkin's disease, as shown in Table 3[19-31]. Moreover, very few data have been published on the therapeutic efficacy of imatinib on lymphoproliferative diseases. Imatinib demonstrated a minimal effect in anaplastic large cell lymphoma with NPM-ALK fusion protein [32] and Hodgkin lymphoma [33] cell lines. In contrast, despite an *in vitro *antiproliferative effect of STI571 on a multiple myeloma cell line [34], no response was observed in a phase II trial of imatinib in patients with refractory/relapsed myeloma [35].

**Table 3:** c-kit expression in lymphoid malignancies

**Type of lymphoma**	**Methods**	**Positive c-kit expression/N (%)**	**References**
Multiple myeloma	Flow cytometry	49/158 (31%)	Kraj 2004
Multiple myeloma	Flow cytometry	17/48 (35%)	Li 2004
Multiple myeloma	Flow cytometry	18/56 (32%)	Ocqueteau 1996
Multiple myeloma	Immunohistochemistry	5/31 (16%)	Potti 2002
Multiple myeloma	Immunohistochemistry	2/72 (3%)	Lugli 2004
Cutaneous plasmacytoma	Immunohistochemistry	13/13 (100%)	Bayer-Garner 2003
**Total MM**	**/**	**104/378 (27%)**	**/**
Lymphoplasmacytic	Immunohistochemistry	0/10 (0%)	Lugli 2004
Lymphoplasmacytic	Flow cytometry	0/7 (0%)	Kraj 2004
Mantle cell L.	Immunohistochemistry	2/17 (1%)	Potti 2002
Lymphomatoid papulosis	Immunohistochemistry	0/18 (0%)	Rassidakis 2004
DLBCL	Immunohistochemistry	24/65 (37%)	Vakiani 2005
**Total B-cell L**.	**/**	**26/117 (22%)**	**/**
CD30+ anaplastic large cell L.	Immunohistochemistry	7/18 (39%)	Brauns 2004
CD30+ anaplastic large cell L.	Immunohistochemistry	11/16 (69%)	Pinto 1994
CD30+ anaplastic large cell L.	Immunohistochemistry	1/78 (1%)	Rassidakis 2004
Lymphomatoid papulosis	Immunohistochemistry	0/18 (0%)	Rassidakis 2004
**Total CD30+ L**.	**/**	**19/130 (15%)**	**/**
Nasal NK/T-cell L.	Immunohistochemistry	0/36 (0%)	Li 2006
Cutaneous T-cell L.	Immunohistochemistry	2/8 (25%)	Brauns 2004
Mycosis fungoides	Immunohistochemistry	6/18 (33%)	Brauns 2004
Sezary's syndrome	Immunohistochemistry	3/5 (60%)	Brauns 2004
**Total T-cell L**.	**/**	**11/67 (16%)**	**/**
Hodgkin's disease	Immunohistochemistry	11/21 (52%)	Pinto 1994
Hodgkin's disease	Immunohistochemistry	0/87 (0%)	Rassidakis 2004
Hodgkin's disease	Immunohistochemistry	0/342 (0%)	Zimpfer 2004
**Total HD**	**/**	**11/450 (2%)**	**/**

**Abbreviations: **L., lymphoma; MM, multiple myeloma; DLBCL, diffuse large B-cell lymphoma; HD, Hodgkin's disease.

The combination of imatinib and chemotherapy has not yet been reported in the literature. However, despite a large number of *in vitro *studies, very few data are available on *in vivo *effect of combined imatinib and chemotherapeutic agents. These reports evaluated combinations of imatinib and zoledronate [36], troxacitabine [37], epothilone B [38], and paclitaxel [39]. In our study, we showed that the imatinib-induced increase of etoposide AUC enhances the tumor growth inhibition of a human xenografted lymphomatous tumor, but not that of an *in vivo *human small cell lung cancer tumor. This observation argues in favor of an increased *in vivo *efficacy of etoposide combined with imatinib, showing that chemotherapy resistance could be reversed by the combination with imatinib in some cancer situations, such as our EBV-associated B-cell lymphomatous model and several other small cell lung cancer xenografts previously reported [4].

A number of published clinical studies have used various chemotherapeutic regimens in various cancer indications. All these data are summarized in Table 4[40-50]. Because of the heterogeneity of these series, it is impossible to clearly define the impact of these combinations on patient outcome. However, they provide a good overview of the toxicities. Some observed side effects can be mainly attributed to the use of antineoplastic drugs, namely hematologic toxicity, nausea and vomiting, infections, and diarrhea, while other side effects could be attributed to the use of imatinib, such as fatigue, peripheral edema, skin eruptions, neuropathy, and liver dysfunction. Only one phase I study of imatinib mesylate combined with doxorubicin and gemcitabine in patients with small cell lung carcinoma showed a high rate of dose-limiting toxicity that required early discontinuation of the trials [47].

**Table 4:** Toxicities of imatinib combined with chemotherapy in human cancers

**References**	**N**	**Treatment protocol**	**Main toxicities**
40	30	STI 400 mg/d + AraC 20 mg/m^2^/d_15–28_.	* Hematologic. * Nausea-vomiting. * Fatigue. * Abdominal pain. * Edema.
41	22	STI 300–800 mg/d + anagrelide 0.5–4 mg/d.	* Hematologic. * Peripheral edema. * Fatigue. * Skin rash.
42	28	STI 600 mg/d + docetaxel 20–45 mg/m^2^/d_1,8,15,22_.	* Hematologic. * Nausea. * Fatigue.
43	20	STI 400 mg/d + hyper-CVAD.	* Hematologic. * Infections.
44	30	STI 400 mg/d + hydroxyurea 1 g/d.	* Edema. * Abdominal pain.
45	20	STI 400/600 mg/d + daunorubicin 50 mg/m^2^/d_1–3 _+ vincristine 2 mg d_1,8,15,22 _+ prednisolone 60 mg/m^2^/d_1–28 _+ L-asparaginase 4000 U/m^2^/d_17–28_.	* Hematologic. * Hyperbilirubinemia.
46	33	STI 400/500 mg/d + hydroxyurea 1 g/d.	* Hematologic.
47	9	STI 300–400 mg/d + doxorubicin 50–60 mg/m^2^/d_1_.	* Hematologic. * Nausea-vomiting. * Reduction of LVEF. * Fatigue.
47	7	STI 300–400 mg/d + gemcitabine 700–800 mg/m^2^/d_1,8,(15)_.	* Hematologic. * Fatigue.
48	9	* STI 300–600 mg/d + irinotecan 65 mg/m^2 ^d_1,8 _+ cisplatin 30 mg/m^2 ^d_1,8_. * STI 300–800 mg/d + irinotecan 60 mg/m^2 ^d_1,8,15 _+ cisplatin 60 mg/m^2 ^d_1_.	* Hematologic. * Diarrhea. * Thrombosis. * Electrolyte disorder.
49	31	STI 800 mg/d + vincristine 2 mg d_1,8,15,22 _+ dexamethasone 40 mg d_1,2,8,9,15,16,22,23_.	* Hematologic. * Infections. * Neuropathy.
50	80	STI 600 mg/d + cyclophosphamide 1200 mg/m^2^/d_1 _+ daunorubicin 50 mg/m^2^/d_1–3 _+ vincristine 1.3 mg/m^2^/d_1,8,15,22 _+ prednisolone 60 mg/m^2^/d_1–21_.	* Hematologic. * Nausea. * Liver dysfunction.

**Abbreviations: **Hyper-CVAD, cyclophosphamide + vincristine + doxorubicin + dexamethasone + methotrexate + cytarabine; LVEF, left ventricular ejection fraction.

## Conclusion

In summary, imatinib potentiates the effects of etoposide in cancer cells such as lymphoma cell lines. These results therefore show that further prospective phase I and II clinical trials with combinations of etoposide-based chemotherapy and imatinib are therefore warranted in some cancer patients, such as malignant lymphoma patients, with careful toxicologic monitoring.

## Authors' contributions

KR, FL, and SW contributed to the pharmacokinetics experiments. IG, VB, RE, and MG contributed to the *in vivo *experiments. PdC contributed to the c-Kit mRNA expression determination. MFP contributed to the correction of the manuscript. DD and FL contributed to the direction of the experiments. DD conceived of the study. All authors read and approved the final manuscript.
